# ﻿Baetidae (Baetidae, Ephemeroptera) in the Maghreb: state of the art, key, and perspectives

**DOI:** 10.3897/zookeys.1139.94586

**Published:** 2023-01-13

**Authors:** Jean-Luc Gattolliat, Boudjéma Samraoui, Nadhira Benhadji, Lina Kechemir, Sonia Zrelli, Sara El Yaagoubi, Majida El Alami El Moutaouakil, Michel Sartori

**Affiliations:** 1 Museum of Zoology, Palais de Rumine, Place Riponne 6, CH-1014 Lausanne, Switzerland Museum of Zoology Lausanne Switzerland; 2 Department of Ecology and Evolution, Biophore, University of Lausanne, CH-1015 Lausanne, Switzerland University of Lausanne Lausanne Switzerland; 3 Laboratoire de Conservation des Zones Humides, University 8 mai 1945, Guelma, Algeria Université 8 Mai 1945 Guelma Algeria; 4 Department of Biology, University Badji Mokhtar Annaba, Annaba, Algeria University Badji Mokhtar Annaba Annaba Algeria; 5 Laboratoire de Recherche Valorisation des Actions de L’homme Pour la Protection de L’environnement et Application en Santé Publique, University of Tlemcen, BP 119, 13000 Tlemcen, Algeria University of Tlemcen Tlemcen Algeria; 6 Department of Hydrobiology, Institute of Biology, University of Szczecin, Felczaka street 3 c, 71- 412 Szczecin, Poland University of Szczecin Szczecin Poland; 7 Faculté des Sciences agronomiques et des sciences biologiques, University of Mouloud Mammeri, Tizi-Ouzou, Algeria University of Mouloud Mammeri Tizi-Ouzou Algeria; 8 Unit of Hydrobiology, Laboratory of Environment Biomonitoring (LBE), Faculty of Sciences of Bizerta, University of Bizerta, 7021 Jarzouna, Tunisia University of Bizerta Jarzouna Tunisia; 9 Honoris United Universities, Ecole Polytechnique Centrale, Avenue Mohamed V, 1002 Tunis, Tunisia Honoris United Universities Tunis Tunisia; 10 Laboratory of Ecology, Systematics and Conservation of the Biodiversity, department of Biology, Faculty of Science, University of Abdelmalek Essaadi, Avenue Sebta, 93002 Tetouan, Morocco University of Abdelmalek Essaadi Tetouan Morocco

**Keywords:** Algeria, aquatic insects, identification key, mayflies, Morocco, Tunisia

## Abstract

Among mayflies, Baetidae are often considered as easy to recognise at the family level, but difficult to identify at lower level. In several faunistic or ecological studies, the identification remains at the family level; Baetidae are generally considered as widespread and ubiquitous, therefore as poorly informative for ecological studies or bioassessments. Here, a straightforward identification key is offered to larvae of the ten genera of Baetidae reported from Maghreb based on easily observable and understandable characters. The diversity, ecology, and distribution of each taxonomic unit (genera or subgenera) are discussed and the main difficulties for deeper identification are pointed out. Future challenges and remaining taxonomic riddles for Maghrebian Baetidae are detailed.

## ﻿Introduction

Ephemeroptera (mayflies) is a small order of insects with approximately 3700 species. Baetidae are the most diversified family as they encompass approximately one third of generic and specific mayfly diversity (Jacobus et al. 2019). The family is almost worldwide distributed but is mostly diversified in the tropics (Gattolliat and Nieto 2009). Mayflies are merolimnic insects, the larval stage is strictly linked to freshwater habitats while the winged stages are aerial. Imaginal stages are extremely brief and have no functional mouthparts and digestive system. Mayflies are the only insects having an intermediate winged stage between larva and imago, called subimago (Barber-James et al. 2008; Sartori and Brittain 2015).

Mayflies are widely used to assess freshwater quality and global changes in hydrosystems. They are generally very abundant, sensitive to environmental alterations, sufficiently diversified and can be considered as efficient bioindicators if identified at a relevant systematic level (Jacobus et al. 2019). Most Mediterranean rivers and streams, and especially Maghrebian ones, suffer from several threats directly or indirectly linked to human activities. Water abstraction for agriculture and domestic use, water pollution and eutrophication, dam construction and other water regulation, in addition to climate change, have direct severe negative impact on the river ecosystem and on aquatic community composition (Hafiane et al. 2016; Morghad et al. 2019; Zerrouk et al. 2021).

The term Maghreb (Arabic for "the west") refers to the countries of western North Africa. In its traditional sense, the Maghreb includes Morocco, Algeria, and Tunisia. The Maghreb, a biogeographic unit, is distinct from the "Greater Maghreb" or "Great Maghreb", a political and historical entity that additionally includes Libya and Mauritania. As no data and materials are available for Libya, and data is limited to a single short checklist for Mauritania (Fraser 1952), we refrain from including these countries in our study. However, we can assume that they have a very impoverished fauna, mainly covered by our study. Despite important improvements in the last decades, the knowledge of the mayfly fauna of the Maghreb is still incomplete. Historically, Eaton (1899) was the first to establish a list of mayflies for a Maghrebian country. He reported thirteen species from Algeria, including six species of Baetidae, one of them being new to science (Eaton 1899). For one century, little attention was paid to this fauna (Lestage 1925; Navás 1929; Kimmins 1938; Grandi 1951; Verrier 1952), till Thomas and collaborators gave a new impulse to the study of this fauna. They described new species and provided new reports in almost all families. In addition, Thomas (1998) provided a provisional checklist of the mayflies from the Maghreb including 69 species: 41 from Morocco, 50 from Algeria, and 29 from Tunisia. He listed 17 species of Baetidae and considered the report of nine additional species as needing to be confirmed. The checklist was updated by various subsequent contributions including description of new taxa and new reports (see below for the complete reference per country).

The Moroccan mayflies remained practically unknown until the 1970s, since only a few reports were available: five species inventoried by Lestage (1925), then seven other species listed by Navás (1929) and Kimmins (1938). The first faunistic inventory dedicated to this group was carried out by Dakki and El Agbani (1983) who were able to identify 26 species of Ephemeroptera, distributed in the different Moroccan regions. This list was greatly enriched subsequently through hydrobiological studies carried out on various Moroccan streams and rivers (Dakki 1986, 1987; Ouahsine and Lavandier 1988a, b; Qninba et al. 1988; El Agbani et al. 1992; El Alami and Dakki 1998; El Alami et al. 2000; El Bazi et al. 2017; Khadri et al. 2017; Mabrouki et al. 2017; Guellaf et al. 2021). Species new for science were also described (Dakki and Giudicelli 1980; Peters 1980; Thomas and Mohati 1985; Dakki and Thomas 1986; Sartori and Thomas 1986; Thomas and Bouzidi 1986; Thomas et al. 1987, 1992; Thomas and Vitte 1988; Vitte and Thomas 1988a, b; Vitte 1991), but only a few of them were on Baetidae (Thomas and Bouzidi 1986; Thomas et al. 1992). Thus, after the synthesis of these works, Thomas (1998) was able to list 41 species distributed in the different Moroccan massifs. Subsequent studies enabled the discovery of nine additional new species or new reports for Morocco; the specific richness for this country reaches 50 species, half of them being Baetidae (Alba-Tercedor and El Alami 1999; El Alami et al. 2000; Mabrouki et al. 2017; Zerrouk et al. 2021). A complete checklist including the diversity and distribution of all Moroccan mayflies was recently published (El Alami et al. 2022a). In order to assess the impact of climate change, human disturbances and pollution on aquatic macroinvertebrates, studies have been carried out over the different geographical Moroccan areas; the main goal was to evaluate the evolution of the mayfly community between the 1980s and the present days by prospecting selected stations in Haut Sebou (Zerrouk et al. 2021), Moulouya (Mabrouki et al. 2017), Ourika (Zuedzang Abessolo et al. 2021) and Rifian watersheds (El Bazi et al. 2017; Khadri et al. 2017; Guellaf et al. 2021).These recent studies of the main Moroccan watersheds confirms the presence of some species never reported since their original descriptions and increase the known distribution of others. Moreover, the specific diversity could increase with the verification of some doubtful identifications through genetic analysis, and the discovery of new species such as the recent description of *Prosopistomamaroccanum* (El Alami et al. 2022b).

In Algeria, the largest African country, it took decades after the pioneering investigations of Eaton (1899) and Lestage (1925), before significant taxonomical progress was made on mayfly knowledge (Soldán and Thomas 1983a; Gagneur and Thomas 1988; Thomas and Vitte 1988; Thomas 1998). Thomas (1998) provisionally listed 50 species of mayflies from Algeria. This checklist is undeniably valuable, despite confirmatory work remains needed as it likely contained some synonymies and misidentifications. In the last few years, a renewed interest in the taxonomy and ecological determinants of mayfly distribution is noted (Mebarki et al. 2017; Benhadji et al. 2018, 2019, 2020; Kechemir et al. 2020; Samraoui et al. 2021a–d). Systematic surveys by the Laboratoire de Conservation des Zones Humides in Algeria are covering eight regions or river basins (Seybouse River, Rhummel, Wadi El Kebir-East, Collo, Aures, Djurdjura, Tiaret, and the Sahara). Collected data have improved knowledge of the distribution and status of Algerian mayflies and led to the discovery of undescribed species (Samraoui et al. 2021c). In addition, collected data have allowed the elucidation of several mayfly life cycles (Bouhala et al. 2020a, b; Samraoui et al. 2021a, d). Further west, investigations of the mayflies from the Tafna River Basin are still proceeding (Benhadji et al. 2020). With 19 reported species, Baetidae is by far the most diversified but also the most problematic family in Algeria. Recent studies allowed the discovery of several potentially new species of Baetidae, as well as species not previously reported from this country (Benhadji et al. 2020; Samraoui et al. 2021b, c; Kaltenbach et al. 2022).

Mayflies from Tunisia encompass 25 species, 12 of them belonging to Baetidae (Zrelli et al. 2016). Boumaiza and Thomas (1986, 1994, 1995) studied and detailed the distribution and ecology of the different species. *Baetispunicus* was originally described from northern Tunisia (Thomas et al. 1983), but it is now reported from the whole Maghreb (Thomas 1998). More recently, a long-term survey was carried out in northern Tunisia, allowing the report of five additional species for this country (including three species of Baetidae) and the description a new species of *Rhithrogena* (Heptageniidae) (Zrelli et al. 2011a, b, 2012, 2016). The most important streams are in northern Tunisia where all the species occur. Despite corresponding to the 4/5 of the territory, the arid southern area only harbours three species. The Tunisian fauna can be considered as relatively well known; recent surveys did not reveal any new taxon or report (Bennas et al. 2018). As in Algeria and Morocco, the main challenges concern the identification of specimens assigned to widely distributed Western European species. Affinities and biogeographical patterns are discussed (Zrelli et al. 2016), but, here again, they need to be updated in the light of new molecular data. As far as we know, after two decades of important surveys, less attentions are paid nowadays on the mayfly systematics, ecology or monitoring in Tunisia (Bennas et al. 2018).

**Figure 1.**
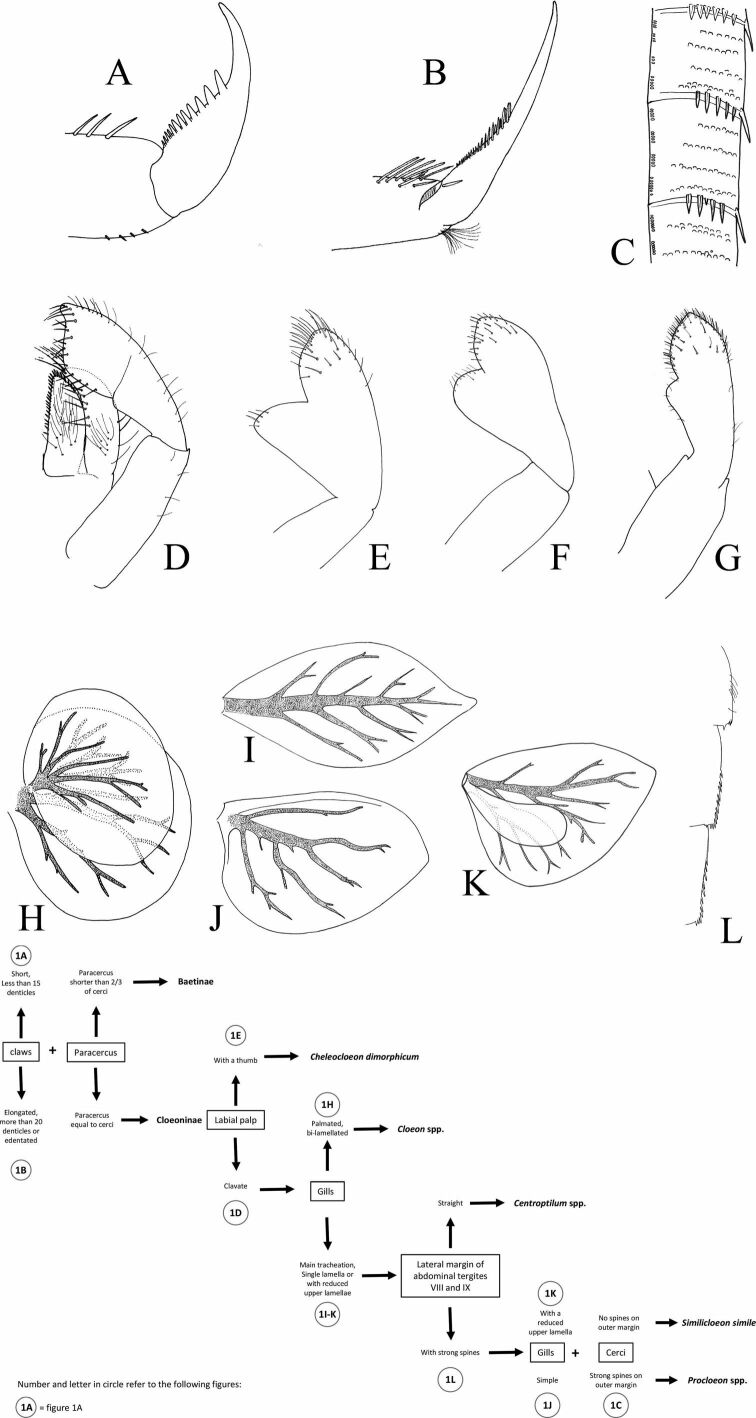
Baetidae: larval characters **A** claw: *Baetis* sp. **B** claw: *Cloeon* sp. **C** lateral margin of cercus: *Procloeon* sp. **D** labial palp: *Cloeonperegrinator***E** labial palp: *Cheleocloeondimorphicum***F** labial palp: Labiobaetiscf.neglectus**G** labial palp: Baetis (Rhodobaetis) sp. **H** abdominal gill IV: *Cloeonperegrinator***I** abdominal gill IV: *Centroptilum* sp. **J** abdominal gill IV: *Procloeon* sp. **K** abdominal gill IV: *Similicloeonsimile***L** lateral margin of abdominal segments VII to IX: *Cloeonperegrinator*.

**Figure 2.**
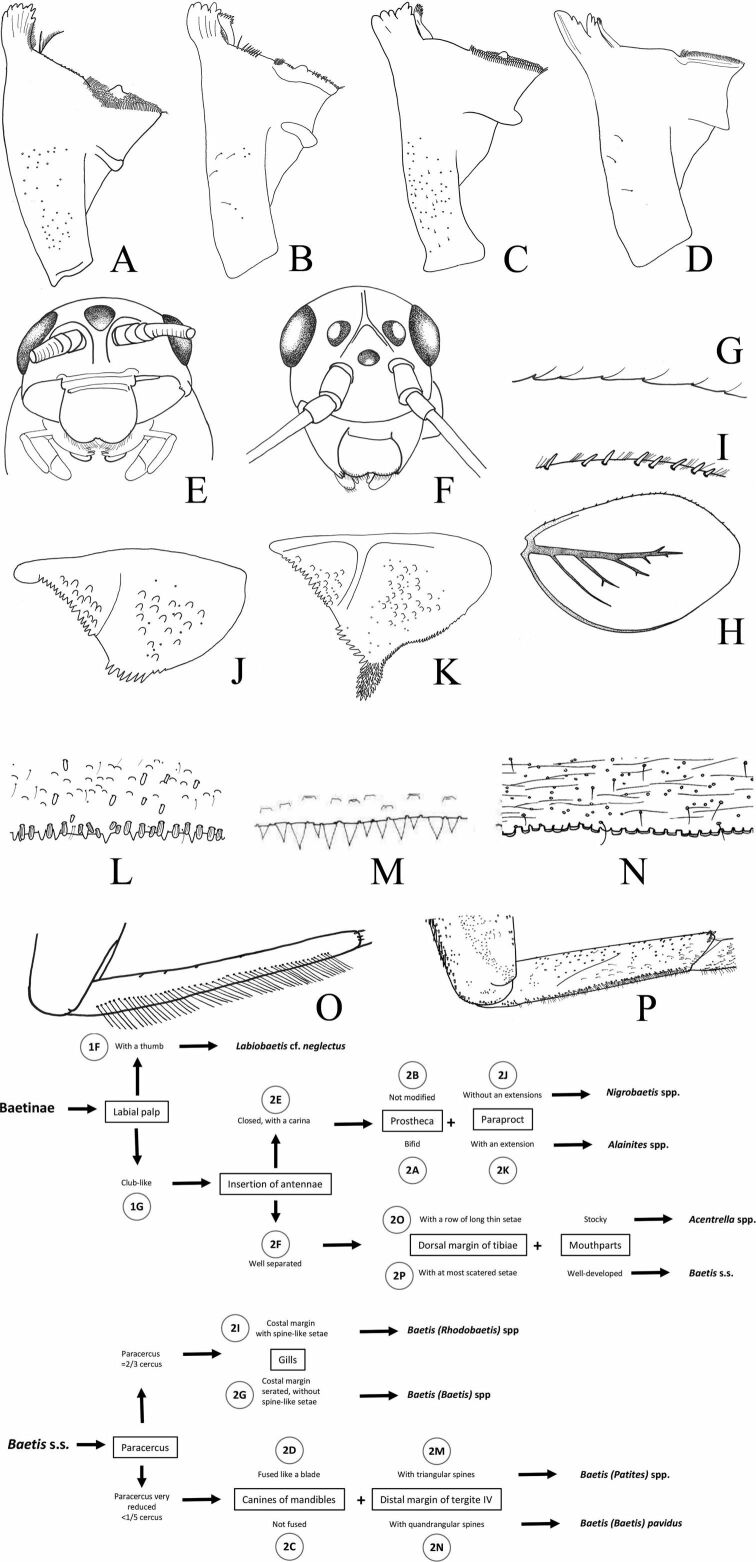
Baetidae: larval characters **A** right mandible: *Alainitessadati***B** right mandible: *Nigrobaetisrhithralis***C** right mandible: Baetis (Rhodobaetis) sp. **D** right mandible: Baetis (Patites) sp. **E** head frontal view: *Alainites* sp. **F** head frontal view: *Baetis* sp. **G** costal margin of gill IV: Baetis (Baetis) sp. **H** gill IV: Baetis (Rhodobaetis) sp. **I** costal margin of gill IV: Baetis (Rhodobaetis) sp. **J** paraproct: *Nigrobaetisrhithralis***K** paraproct: *Alainitessadati***L** distal margin tergite IV: Baetis (Rhodobaetis) sp. **M** distal margin tergite IV: Baetis (Patites) sp. **N** distal margin tergite IV: Baetis (Baetis)pavidus**O** foretibia: Acentrellacf.sinaica**P** foretibia: Baetis (Rhodobaetis) sp.

In Baetidae, imaginal stages remain difficult to identify to the species level. Larvae are easier to determine at a finest taxonomic level. Moreover, they are generally present all-year-round while emergence can be more sporadic. Therefore, collecting larvae generally remains the most efficient method to correctly assess the local fauna. Our main aim is to provide a key as easy to use as possible to allow a secure identification of baetid larvae to the most efficient level. The circled alphanumeric codes (1a, 1b, ...) indicated in the dichotomous key refer to the different illustrations of Figs 1, 2. We also summarize the main difficulties and gaps in knowledge.

## ﻿Materials and methods

Mayfly larvae can be sampled using a Surber net or a dipnet, then stored in alcohol ideally at 80% to 95%. To preserve DNA, they must not be fixed in 5% formaldehyde. Adults can be collected with handnets or attracted by light traps. For the association of the ontogenic stages or just obtaining imagoes, rearing can be made in the field (following detailed instructions presented in http://www.insecta.bio.spbu.ru/z/rearing.htm). Rearing larvae in the laboratory until emergence generally requests equipment for water oxygenation. Association of the different stages can be also securely made by using molecular barcodes (Gattolliat and Monaghan 2010; Gattolliat et al. 2012, 2018).

Identification at the family or generic levels can be generally made under an efficient stereo microscope. In most cases, specific identification request slide mounting and observation under a compound microscope. Dissection can be made in alcohol or in Cellosolve (2-Ethoxyethanol), in adequation with subsequent mounting liquid (Canada balsam or Euparal). Identification to the species level based on mesoscopic characters such as abdominal pattern, shape and setation on legs, relative length of cerci and paracercus or tergite ornamentation should be restricted to the case of well-known fauna of a restricted watershed with examiners possessing important skills and training.

DNA can be extracted from the whole specimens or just from a small part, such as leg or thorax; specimens must be stored in alcohol at high concentration, without denaturant. Long term storage under inappropriate conditions (high temperature or temperature variations) may fragment DNA and inhibit the gene amplification. Non-destructive methods allowing subsequent morphological analysis should be preferentially used (see Vuataz et al. (2011) for details). For routine procedure, most effective results are obtained by amplifying the 658 bp fragment of the mitochondrial gene cytochrome oxidase subunit 1 (COI) using the primers LCO 1490 and HCO 2198 (Folmer et al. 1994, see Kaltenbach et al. (2020) for details).

## ﻿Results

### ﻿Key

Figs 1, 2

### ﻿Synopses of genera

#### ﻿1. Acentrella

Taxon classificationAnimaliaEphemeropteraBaetidae

Bengtsson, 1912

502C50B3-20EF-56B4-B91A-8C3B5B7FC995

##### Diagnosis.

1) Very reduced paracercus; 2) stocky mouthparts; 3) head compressed dorsoventrally; 4) presence of a complete row of long thin setae on the dorsal margin of tibia; 5) villopore present on the ventral margin of fore femora.

##### Remarks.

**Figure 3.**
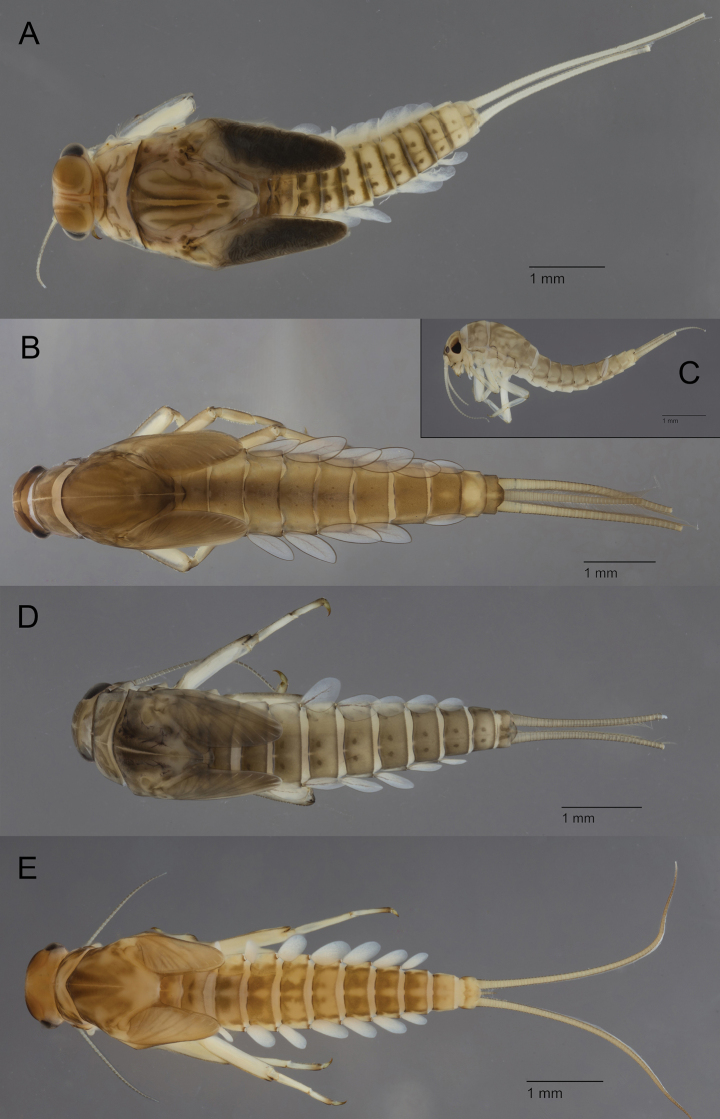
Baetidae: larvae in toto in dorsal view (except 3C lateral view) **A**Acentrellacf.sinaica**B***Alainitesoukaimeden***C***Alainitessadati***D**Baetis (Patites) sp. **E**Baetis (Baetis) pavidus.

In the past, *Acentrella* was considered as a subgenus of *Baetis* (Müller-Liebenau 1969). Confusions with species with reduced paracercus (*Baetispavidus* or the subgenus Patites) can be avoided by the examination of the mouthparts, especially of the mandibles as well as the distal margin of the tergites. The abdominal tergites also present a characteristic dark brown pattern (Fig. 3A).

Two species of *Acentrella* are reported in the Maghreb: Acentrellacf.sinaica Bogoescu, 1931 and *Acentrellaalmohades* Alba-Tercedor & El-Alami, 1999. *Acentrellasinaica* was originally described from Romania, then reported from several countries from Central and South Europe. This species is not abundant but widely distributed in North Algeria and North-West Tunisia. Maghrebian populations seem morphologically very similar to those from central Europe. However, molecular preliminary results suggest that the Maghreb populations most probably belong to a new undescribed species (Benhadji et al. 2020). *Acentrellaalmohades* is originally described from the Rif mountains and middle Atlas as well as from south-west of Spain. The two species do not seem to co-occur. They can be separated by the length of the setae of the dorsal margin of femora (longer in A.cf.sinaica than in *A.almohades*) and the number of regular rows of stout setae at apex of paraglossa (three rows in A.cf.sinaica, four rows in *A.almohades*) (Alba-Tercedor and El-Alami 1999).

#### ﻿2. Alainites

Taxon classificationAnimaliaEphemeropteraBaetidae

Waltz & McCafferty, 1994

40FA9908-41FF-56B2-8905-9090862C031A

##### Diagnosis.

1) Antennae located close together with a well-developed carina in between; 2) paraproct with unique elongate prolongation on distal margin; 3) mouthparts in a hypognathous position giving to the body a characteristic curved posture in lateral view; 4) right mandible with a bifid prostheca.

##### Remarks.

The position of the antenna and the general posture in lateral view (Fig. 3C) easily separate *Alainites* and *Nigrobaetis* from other genera in the Maghreb. The prolongation of the distal margin of paraproct (Fig. 2K) and the bifid prostheca (Fig. 2A) unambiguously separate *Alainites* from all the other Maghreb genera including *Nigrobaetis*.

Three species of *Alainites* are reported in the Maghreb: Alainitescf.muticus (Linnaeus, 1758), *Alainitesoukaimeden* (Thomas & Sartori, 1992) (Fig. 3B) and *Alainitessadati* Thomas, 1994 (Fig. 3C). *Alainitesoukaimeden* and *A.sadati* are endemic to the Maghreb and present allopatric distribution: *A.sadati* is widely distributed in north Algeria and north Tunisia (Zrelli et al. 2012), while *A.oukaimeden* is only reported from the High Atlas, Morocco (Thomas et al. 1992; El Alami et al. 2022a). The two endemic species have six pairs of gills; they can be only separated by intricate characters such as the reticulation of the surface of tergites and mandibles, the shape of the spines of distal margin of tergite IV and the number of strong setae on the dorsal margin of fore femora. A still undescribed new species, closely related to the West Palaearctic species *Alainitesmuticus*, with seven pairs of gills, is present in Maghreb but with a restricted distribution limited to northern Morocco (El Alami et al. 2022a).

#### ﻿3. Baetis

Taxon classificationAnimaliaEphemeropteraBaetidae

Leach, 1815

E5C03274-84BC-5E3A-941C-8F3273417CB0

##### Diagnosis.

1) Claw short generally with a single row of restricted number of denticles (exception *B.maurus* with two rows of denticles); 2) paracercus reduced or at most equal to 2/3 of the cerci; 3) presence of a villopore on the ventral margin of fore femora; 4) mouthparts normally developed.

##### Remarks.

Except for the presence of the villopore (which is also present in *Acentrella* and *Labiobaetis*), the genus *Baetis* is mainly defined by the absence of characters. The genus encompasses three subgenera in the Maghreb. These subgenera are relatively easy to recognize and must be considered as the suitable identification level to reach. Except for a few cases, species identification is rather difficult and requires expertise.

#### ﻿3.1 Baetis (Baetis)

Taxon classificationAnimaliaEphemeropteraBaetidae

1BD90644-5734-5F43-AA64-E4981068ADC8

##### Diagnosis.

1) Canines of the right and left mandibles not fused and not forming a blade-like tooth; 2) costal margin of gills serrated but without spine-like setae; 3) distal margin of tergites with triangular or quadrangular spines but without spatulas.

##### Remarks.

As for the genus, the nominal subgenus Baetis is mostly defined by the absence of unique characters (mouthparts and legs not modified). Baetis (Baetis) pavidus Grandi, 1949 (Fig. 3E), described from Italy, is by far the most common species of Baetidae in lower and middle section of streams and rivers. The Maghreb populations are morphologically extremely close to European ones. Moreover, from a genetic point of view, they belong to the same species as populations from Spain and South of France (Benhadji et al. 2020). No sequences are, for the moment, available from continental Italy. This species seems to be rare and restricted in Italy and South of France, while it is the most successful species in the Maghreb. It can be recognised by the very short paracercus and the distal margin of the tergites with quadrangular spines. Presence of other species of the subgenus Baetis is certain at least in Morocco, but the species identification remains problematic. Baetis (Baetis) fuscatus (Linnaeus, 1760), Baetis (Baetis) meridionalis Ikonomov, 1954, and Baetis (Baetis) nigrescens Navás, 1932 were reported from Morocco (Thomas 1998; El Alami et al. 2000); but it remains unclear if they really occur in this region or if these reports represent in fact either new species or more recently described species.

#### ﻿3.2 Baetis (Patites)

Taxon classificationAnimaliaEphemeropteraBaetidae

Thomas & Dia, 2000

FA55AA4E-D0FD-5397-BFD1-3280891557E3

##### Diagnosis.

1) paracercus reduced to a few segments; 2) labrum rectangular with a row of numerous setae parallel to the distal margin; 3) canines of the right and left mandibles fused to form a blade-like tooth; 4) distal margin of tergite IV with triangular spines.

##### Remarks.

The subgenus Patites was initially established for *Baetismelanonyx* and related species (Thomas and Dia 2000). The present concept of the subgenus encompasses all the species previously assigned to the *alpinus* species group (sensu Müller-Liebenau 1969), despite most of the species were never formally transferred to this subgenus. This subgenus encompasses at least three species in the Maghreb: Baetis (Patites) berberus Thomas, 1986, Baetis (Patites) maurus Kimmins, 1938, and Baetis (Patites) punicus Thomas, Boumaiza & Soldán, 1983. All of them have two dark spots on each abdominal tergite (Fig. 3D). Baetis (Patites) maurus is the only species of *Baetis* s. l. with two rows of denticles on all claws (Soldán and Thomas 1983a; Thomas et al. 1983). This character allows an easy and unambiguous identification of the species in the Maghreb. Baetis (Patites) berberus and Baetis (Patites) punicus are much more difficult to identify with confidence; especially as the preliminary molecular results indicate that *Patites* is much more diversified than expected and new sibling species are expected (Murria et al. 2017; Benhadji et al. 2020).

#### ﻿3.3 Baetis (Rhodobaetis)

Taxon classificationAnimaliaEphemeropteraBaetidae

Jacob, 2003

09D68C63-2470-5CF7-8AAE-5DF82F0F33FF

##### Diagnosis.

1) Gills with spine-like setae along the costal margin; 2) Distal margin of tergites with spatulas in addition to triangular spines; 3) paracercus length 2/3 of cerci.

##### Remarks.

**Figure 4.**
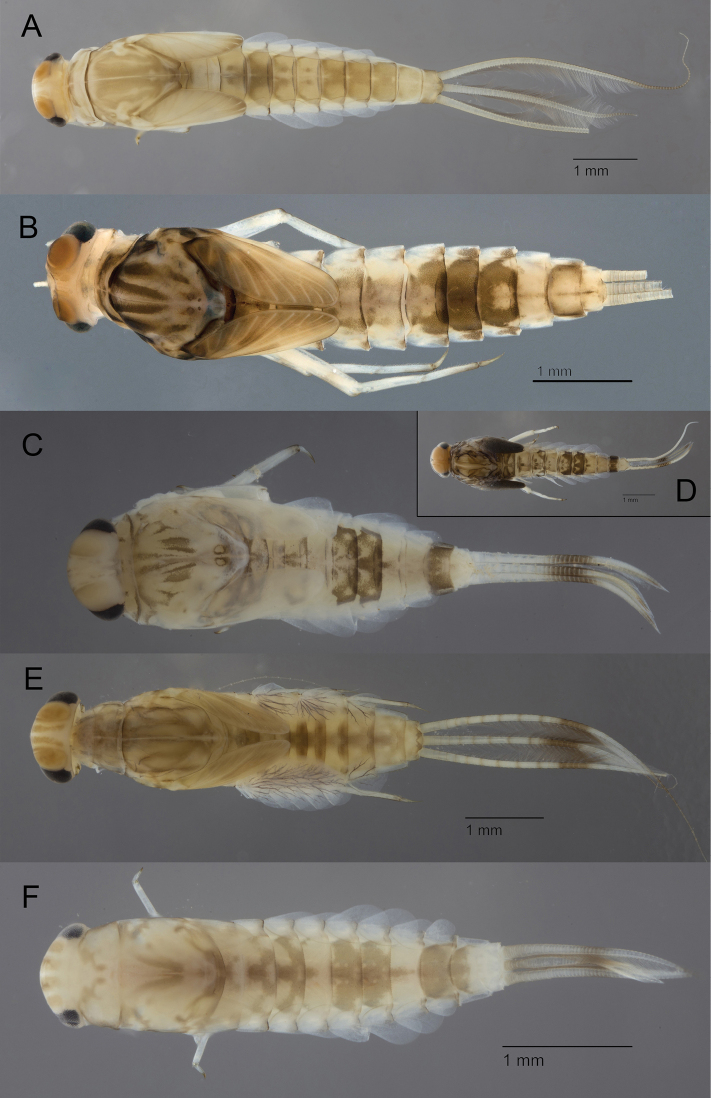
Baetidae: larvae in toto in dorsal view **A**Baetis (Rhodobaetis) atlanticus**B***Centroptilumalamiae***C***Cheleocloeondimorphicum***D***Cheleocloeondimorphicum* (ready to molt specimen) **E***Cloeonperegrinator***F**Labiobaetiscf.neglectus.

The subgenus Rhodobaetis is widely distributed in the Maghreb where it colonizes all types of running waters. Colouration, size, setation of legs and degree of development of the spine-like setae on the gills are highly variable, but may also represent plasticity and intraspecific variations. Three species of *Rhodobaetis* are reported from Maghreb with certainty: Baetis (Rhodobaetis) atlanticus Soldán & Godunko, 2006 (Fig. 4A), Baetis (Rhodobaetis) chelif Soldán, Godunko & Thomas, 2005 and Baetis (Rhodobaetis) sinespinosus Soldán & Thomas, 1983. Reports of Baetis (Rhodobaetis) rhodani (Pictet, 1843) in Maghreb probably concern misidentification of one of the three species mentioned above. In most cases, B. (R.) rhodani must be considered sensu lato and by consequence as equivalent to *Rhodobaetis*. Distinction of the three species is rather difficult as important intraspecific variations have been found at least in B. (R.) atlanticus. Only two reliable characters allow the separation of the three species: B. (R.) sinespinosus has no scale at the tip of maxillary palp and four rows of setae at apex of paraglossae; B. (R.) atlanticus and B. (R.) chelif have one scale at the tip of maxillary palp and differ by number of rows at the apex of paraglossae (three in B. (R.) atlanticus and four in B. (R.) chelif) (Soldán and Thomas 1983a; Soldán et al. 2005; Soldán and Godunko 2006). The three species are at least partially sympatric and can be collected in the same site. Specific identification is therefore very difficult. It requires high expertise and slides preparation; it should be also corroborated by molecular analysis.

#### ﻿4. Centroptilum

Taxon classificationAnimaliaEphemeropteraBaetidae

Eaton, 1869

0E232ED6-70BE-55B2-A0A3-63F397638AC9

##### Diagnosis.

1) Both mandibles with a row of abundant setae between prostheca and mola; 2) gills present on segment I to VII, all simple and elongated; 3) absence of spines on the lateral margin of abdominal segments; 4) paracercus subequal in length to cerci.

##### Remarks.

All the specimens we checked from the North-East of Algeria and North Morocco belong to the two recently described species *Centroptilumalamiae* Kaltenbach, Vuataz & Gattolliat, 2022 (Fig. 4B) and *Centroptilumsamraouii* Kaltenbach, Vuataz & Gattolliat, 2022. Both species are closely related to *Centroptilumluteolum* (Müller, 1776) but clearly different both morphologically and genetically (Kaltenbach et al. 2022). The description of the species *Centroptilumalgiricum* Eaton, 1899 was based on male and female imagoes collected close to Tizi-Ouzou (Algeria) (Eaton 1899). According to the shape of the hindwing, especially of its apex, this species should be assigned to *Procloeon* rather than to *Centroptilum* and therefore cannot be considered as the winged stage of one of the two new species of *Centroptilum* (Samraoui et al. 2021c).

#### ﻿5. Cheleocloeon

Taxon classificationAnimaliaEphemeropteraBaetidae

Wuillot & Gillies, 1993

BB8988B9-0287-58E3-873D-2B3A1D02ACFC

##### Diagnosis.

1) Claws elongate with two rows of minute denticles; 2) Second segment of the labial palp with a thumb-like process; 3) paracercus subequal to cerci; 4) male with hindwing pads and female without.

##### Remarks.

The genus *Cheleocloeon* is mostly diversified in the Afrotropical region and reaches in the Maghreb its north-western limit. *Cheleocloeondimorphicum* (Soldán & Thomas, 1985) is the single species of the genus reported from Maghreb (Fig. 4C, D). This endemic species is widespread through this area but seems nowhere abundant (Soldán and Thomas 1985; Boumaiza and Thomas 1995; Mabrouki et al. 2017; El Alami et al. 2022a).

#### ﻿6. Cloeon

Taxon classificationAnimaliaEphemeropteraBaetidae

Leach, 1815

982677AB-154F-517B-BA1A-30C463527C27

##### Diagnosis.

1) Gills I-VI with double lamellae, upper lamellae similar in shape to lower ones and only slightly smaller; 2) legs elongated, claw elongated with two rows of abundant small to medium denticles; 3) labial palp conical and truncated; 4) maxillary palp 3-segmented; 5) paracercus subequal in length to cerci.

##### Remarks.

*Cloeon* is the most common and most abundant mayfly genus in still and standing waters all over the world (except in America). It can survive in conditions with high temperature and very low oxygen level. Cloeongr.dipterum is a complex of very similar species. In Maghrebian ecological and faunistic surveys, it is generally referred as *Cloeondipterum* (Linneaus, 1761) or *Cloeoncognatum* Stephens, 1835 (Boumaiza and Thomas 1995; Thomas 1998; Mabrouki et al. 2017; El Alami et al. 2022a). Recent molecular studies support the presence of six lineages in the West Palearctic and at least one of them is present in the Maghreb (Rutschmann et al. 2014, 2017). This lineage corresponds to *Cloeonperegrinator* Gattolliat & Sartori, 2008 (Fig. 4E), a species originally thought to be endemic to Macaronesian archipelago but reported later from Algeria (Gattolliat et al. 2008; Benhadji et al. 2020). Cloeongr.dipterum is known to present high plasticity; for example the size of the gills is directly adapted to the concentration of dissolved oxygen (Sweeney et al. 2018). Therefore, for the moment, identification to the species level can only be securely made based on molecular evidence (CO1 barcoding).

Besides Cloeongrdipterum, another species, *Cloeonsaharense* Soldán & Thomas, 1983, was reported from different localities in intermittent brooks and pools in arid and subarid zones of Algeria (Soldán and Thomas 1983a). This species should be easily separated from C.gr.dipterum by the absence of spines on the lateral side of abdominal segments, a character which is unique among *Cloeon*. Forewing of female imagoes are hyaline while those of C.gr.dipterum have costal and subcostal areas with dark brown pattern (Soldán and Thomas 1983a). Although this species is supposed to be morphologically easily recognisable, *C.saharense* has never been reported from the Maghreb since its original description.

#### ﻿7. Labiobaetis

Taxon classificationAnimaliaEphemeropteraBaetidae

Novikova & Kluge, 1987

DEB13D4F-F3E4-5514-8D4C-0B56B33D1404

##### Diagnosis.

1) Claws hooked with one row of well-developed denticles; 2) second segment of the labial palp with a thumb-like process; 3) paracercus 2/3 of cerci.

##### Remarks.

All the Maghreb specimens of *Labiobaetis* were assigned to the Iberian species *Labiobaetisneglectus* (Navàs, 1913) (Fig. 4F). Originally the species was only described at the imaginal stage. The type material is lost, and the original description is very succinct. The specific attribution of the specimens from Algeria to *L.neglectus* was based on rather obscure criteria (Soldán and Thomas 1983a). In the same publication, the authors provided the first description of the larval stage based on material from Algeria. Subsequent reports of the species only concerned larvae (Zrelli et al. 2016; Mabrouki et al. 2017; Samraoui et al. 2021c; El Alami et al. 2022a), and were only based on the characters depicted by Soldán and Thomas (1983a). Examination of larvae from the type locality in Spain is a crucial point to confirm or refute the conspecificity of Maghrebian and Iberian populations.

#### ﻿8. Nigrobaetis

Taxon classificationAnimaliaEphemeropteraBaetidae

Novikova & Kluge, 1987

6017C9A8-89FB-59F4-B135-7237C9298FAC

##### Diagnosis.

1) Antennae located close together with a well-developed carina in between; 2) mouthparts in a hypognathous position giving to the body a curved posture in lateral view; 3) right mandible with a simple robust prostheca; 4) paraproct without protuberance.

##### Remarks.

The position of the antenna and the general posture in lateral view easily separate *Alainites* and *Nigrobaetis* from other genera in Maghreb. Contrary to *Alainites*, *Nigrobaetis* presents unmodified paraproct (Fig. 2J) and prostheca (Fig. 2B).

**Figure 5.**
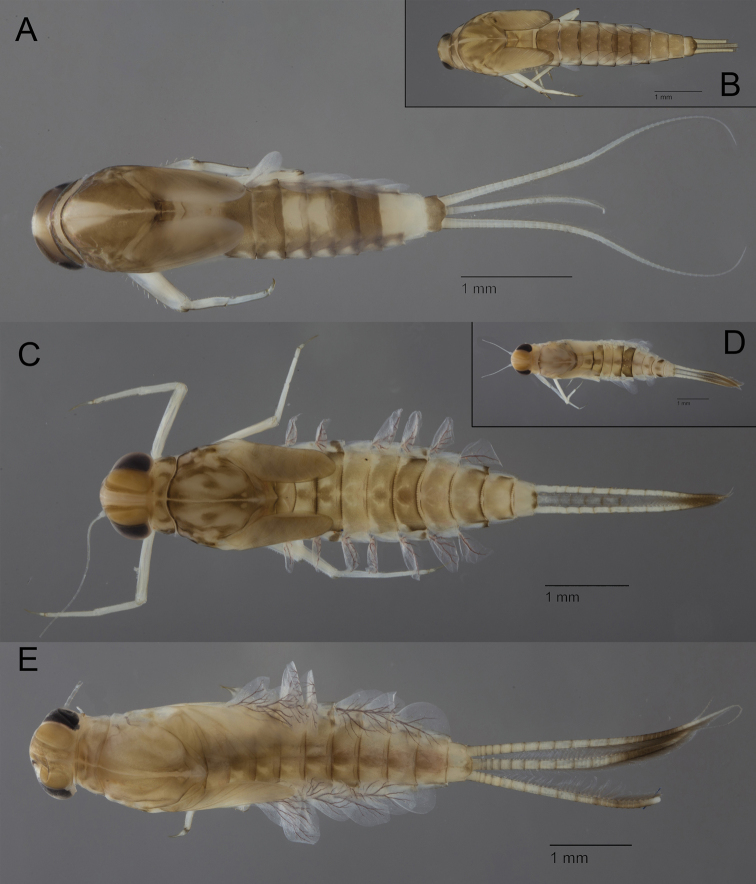
Baetidae: larvae in toto in dorsal view **A***Nigrobaetisnumidicus***B***Nigrobaetisrhithralis***C***Procloeonstagnicola***D**Procloeoncf.pennulatum**E***Similicloeonsimile*.

Two species of *Nigrobaetis* are reported in the Maghreb: *Nigrobaetisnumidicus* (Soldán & Thomas, 1983) (Fig. 5A) and *Nigrobaetisrhithralis* (Soldán & Thomas, 1983) (Fig. 5B). *Nigrobaetisrhithralis* is widely distributed through the Maghreb from Tunisia to Morocco but is rather restricted and never abundant (El Alami et al. 2000; Godunko et al. 2018).

*Nigrobaetisnumidicus* was originally described from Oued Chiffa, close to Alger at an altitude of 200m. It was most certainly a very rare species there, as only four specimens were collected (Soldán and Thomas 1983b). Despite being easily recognizable by the smooth distal margin of abdominal tergites and its peculiar tergal pattern (Fig. 5A), this species was never reported from Algeria since its original description; in Morocco it seems to only occur in a few localities of the Middle Atlas and Rif (Zerrouk et al. 2021; El Alami et al. 2022a).

#### ﻿9. Procloeon

Taxon classificationAnimaliaEphemeropteraBaetidae

Bengtsson, 1915

2C2047A4-CC75-51DE-8209-436C1A3EBF4E

##### Diagnosis.

1) Gills I-VI with simple or double lamellae, if double, the upper lamella much smaller than lower lamella; 2) legs elongated, claw elongated with two rows of small to minute denticles; 3) labial palp conical and truncated; 4) lateral margin of abdominal segments VII–IX with strong spines; 5) paracercus subequal in length to cerci; 6) cerci with strong spines on the outer margin.

##### Remarks.

*Procloeonstagnicola* Soldán & Thomas, 1983 (Fig. 5C) is rather similar to the European species *Procloeonbifidum* (Bengtsson, 1912). Old reports of *P.bifidum* most certainly refer to *P.stagnicola*. This species possesses gills with single lamellae (Soldán and Thomas 1983a). It is widely distributed in the Maghreb. Besides this endemic species, reports of other species of the genus are more problematic. Reports of *Procloeonpennulatum* (Eaton, 1870) are limited to Morocco (Fig. 5D). Within this species, morphological comparison between Maghrebian and Central European specimens was still not performed, and no molecular analyses are available. The conspecificity needs to be confirmed by morphological and molecular evidence; the identification is probably mostly based on presence of hindwings and very long claws. *Procloeonconcinnum* (Eaton, 1885) was originally described from Portugal and is only known at the imaginal stage: eggs, larvae and subimagoes remain unknown (Bauernfeind and Soldán 2012). It is unclear which characters allow a reliable assignment of specimens from Morocco to this species, especially at the larval stage (El Bazi et al. 2017; Khadri et al. 2017; Mabrouki et al. 2017; Guellaf et al. 2021). As mentioned above, *Centroptilumalgiricum* Eaton, 1899 should be assigned to *Procloeon* based to the shape of the hindwing, and may be the imaginal stage of one of the known species of *Procloeon* (Samraoui et al. 2021c).

#### ﻿10. Similicloeon

Taxon classificationAnimaliaEphemeropteraBaetidae

Kluge & Novikova, 1992

08195762-0B78-5214-8703-69C91C9BB479

##### Diagnosis.

1) Gills I–VI with double lamellae, upper lamella much smaller than lower lamella; 2) legs elongated, claw elongated with two rows of small to minute denticles; 3) labial palp conical and truncated; 4) maxillary palp 2-segmented; 5) paracercus subequal in length to cerci; 6) lateral margin of abdominal segments VII to IX with strong spines; 6) cerci without spines on the outer lateral margin.

##### Remarks.

*Similicloeon* present intermediate characters between *Cloeon* and *Procloeon*; it may be confused with either of them. It was first considered as a subgenus of *Cloeon* and was only recently raised to the generic level (Kluge and Novikova 1992; Kluge 2016). *Similicloeonsimile* (Eaton, 1870) is the single species of the genus known from the Maghreb (Fig. 5E). No morphological differences or genetic distances were observed between Maghrebian and Central European populations (unpublished data). A restricted part of the reports of *Cloeon* sp. may represent misidentification of *S.simile* (as in most previous keys, *Similicloeon* is not separated from *Cloeon*). This species is rarely reported but seems rather widespread across the region (Boumaiza and Thomas 1995; Khadri et al. 2017; Mabrouki et al. 2017). This species is considered as highly euryhaline (Boumaiza and Thomas 1995). Based on the variety of colonized habitats, Mabrouki et al. (2017) suggested that the genus is probably not monospecific in Morocco; we have no evidence to confirm or refute this hypothesis.

## ﻿Checklist of valid species

*Acentrellaalmohades* Alba-Tercedor & El-Alami, 1999

Acentrellacf.sinaica Bogoescu, 1931

Alainitescf.muticus (Linnaeus, 1758)

*Alainitesoukaimeden* (Thomas & Sartori, 1992)

*Alainitessadati* Thomas, 1994

Baetis (Baetis) cf. fuscatus (Linnaeus, 1760)

Baetis (Baetis) pavidus Grandi, 1949

Baetis (Patites) berberus Thomas, 1986

Baetis (Patites) maurus Kimmins, 1938

Baetis (Patites) punicus Thomas, Boumaiza & Soldán, 1983

Baetis (Rhodobaetis) atlanticus Soldán & Godunko, 2006

Baetis (Rhodobaetis) chelif Soldán, Godunko & Thomas, 2005

Baetis (Rhodobaetis) sinespinosus Soldán & Thomas, 1983

*Centroptilumalamiae* Kaltenbach, Vuataz & Gattolliat, 2022

*Centroptilumsamraouii* Kaltenbach, Vuataz & Gattolliat, 2022

*Cheleocloeondimorphicum* (Soldán & Thomas, 1985)

*Cloeonperegrinator* Gattolliat & Sartori, 2008

*Cloeonsaharense* Soldán & Thomas, 1983

Labiobaetiscf.neglectus (Navàs, 1913)

*Nigrobaetisnumidicus* (Soldán & Thomas, 1983)

*Nigrobaetisrhithralis* (Soldán & Thomas, 1983)

*Procloeonalgiricum* (Eaton, 1899)

Procloeoncf.pennulatum (Eaton, 1870)

*Procloeonstagnicola* Soldán & Thomas, 1983

*Similicloeonsimile* (Eaton, 1870)

### Reported species with uncertain status

Baetis (Rhodobaetis) rhodani (Pictet, 1843)

Baetis (Baetis) meridionalis Ikonomov, 1954

Baetis (Baetis) nigrescens Navás, 1932

*Cloeondipterum* (Linneaus, 1761)

*Cloeoncognatum* Stephens, 1835

*Procloeonbifidum* (Bengtsson, 1912)

*Procloeonconcinnum* (Eaton, 1885)

## ﻿Discussion

In the Maghreb, Baetidae are the most diversified family of mayflies; they encompass ten genera, and three subgenera. We offer a straightforward dichotomic key to separate this family in twelve taxonomic units corresponding either to genera or subgenera. In the future, these taxonomic units should represent the standard identification level for environmental studies and water quality assessment. Among the 25 species of Baetidae reported from Maghreb, at least fourteen species are endemic to this area, underlying the diversity and uniqueness of this fauna. With eight species, *Baetis* is by far the most species-rich genus; other genera only contain one or two species. Links between European and Maghrebian faunas exist (Thomas 1998; Zrelli et al. 2016) but are less important than previously thought (Benhadji et al. 2020). Only six species unambiguously occur in the Maghreb and in Central Europe. *Similicloeonsimile* is widely distributed in West Palearctic and in the Maghreb; *Cloeonperegrinator* is reported from Macaronesia and the Maghreb; *Baetisatlanticus* was originally described from Madeira, it is now reported from the Maghreb to Sweden including the British Islands; *Baetispavidus* is extremely common in the whole Maghreb but seems rare in South of France and Italy; *Acentrellaalmohades* and *Baetismaurus* occur in the Maghreb and the Iberian Peninsula (Alba-Tercedor and El Alami 1999; Gattolliat et al. 2008; Benhadji et al. 2020; Samraoui et al. 2021b; El Alami et al. 2022a). Five taxa are tentatively attributed to Central European species (indicated as cf. in the list). Preliminary studies indicated that Acentrellacf.sinaica, Alainitescf.muticus and Labiobaetiscf.neglectus morphologically and/or genetically differ between these two regions (Benhadji et al. 2020; Samraoui et al. 2021c). These three taxa may represent endemic species to the Maghreb, closely related to their European sister species. The case of Cloeoncf.dipterum is more problematic as the species concept itself remains unclear. This complex of species encompasses at least six different lineages in the West Palearctic based on molecular evidence only, representing the same number of potential species (Rutschmann et al. 2014, 2017). According to our present knowledge, it remains impossible to decide which lineage corresponds to *Cloeondipterumsensu stricto.* For the moment, only one lineage is reported with certainty from the Maghreb and it corresponds to *Cloeonperegrinator* (Benhadji et al. 2020). According to the diversity of habitats colonized by the larvae and the important morphological differences observed between populations, we could assume that more than one species occurs in the Maghreb. Species identification within *Cloeon* remains impossible without a broad scale study based on an integrative approach.

We consider as dubious, or at least requiring confirmation, the reports of seven species in the Maghreb, all of them having a European distribution. *Baetisrhodani*, *Cloeondipterum*, and *Cloeoncognatum* belong to complexes of very close species; other species from these complexes are already reported from the Maghreb. However, the presence of these three species cannot be completely excluded. At least an important part of their reports corresponds to old identifications and are based on inappropriate concepts. According to preliminary results (El Alami et al. 2022a), some specimens of *Baetis* from Morocco cannot be assigned to any reported species. They clearly belong to the subgenus Baetis, their paracercus is not reduced and they do not exhibit spines on the margin of gills. They cannot be assigned to any species of the checklist; they are reported either as Baetisgr.fuscatus or Baetisgr.lutheri in El Alami et al. (2022a). As mentioned above, beside *P.stagnicola*, other species of *Procloeon* need a complete revision and extensive morphological and molecular comparisons with Iberian and Central European populations.

All these problematic cases clearly indicate the need of an extensive taxonomic revision in some taxa including specimens from Maghrebian, Mediterranean and Central European populations. Only an integrative approach involving at least morphology and molecular evidence can solve these taxonomic riddles.

In conclusion, Baetidae is the most diverse family of mayflies in the Maghreb. This family encompasses on the one hand common species with large ecological valence (e.g., *Baetispavidus*, *Baetisatlanticus*, *Cloeonperegrinator*) and, on the other hand, rare species with very specific ecological requirements (e.g., *Nigrobaetisrhithralis*, *Nigrobaetisnumidicus*, *Alainitessadati*). Therefore, identification to the family level may completely hide important environmental information as key conservation values. By offering a reasonably simple key to generic or subgeneric level, the main goal is to open the identification of this family to a wide range of scientists, and not only to a restricted set of experts. We hope that further ecological or environmental studies will confirm the high potential of this group for bioindication when working at lower taxonomic level. A better understanding of the distribution and ecology of the members of this family is an essential step for the conservation of these species and of the endangered freshwater habitat in general.

## Supplementary Material

XML Treatment for Acentrella

XML Treatment for Alainites

XML Treatment for Baetis

XML Treatment for Baetis (Baetis)

XML Treatment for Baetis (Patites)

XML Treatment for Baetis (Rhodobaetis)

XML Treatment for Centroptilum

XML Treatment for Cheleocloeon

XML Treatment for Cloeon

XML Treatment for Labiobaetis

XML Treatment for Nigrobaetis

XML Treatment for Procloeon

XML Treatment for Similicloeon
